# 

**Published:** 1843-03-04

**Authors:** 


CONTENTS.

A Course of Clinical Lectures, delivered at the Hôtel-Dieu, Paris, for the Session 1842–'43. By A. F. Chomel. Lecture 3.

Case 1. Phthisis, complicated with ascites and œdema of the lower extremities — Anatomical lesions corresponding to these phenomena, . 37

Case 2. Pleurodynia — Necessity of combatting promptly and energetically pleurodynic pains in order to avoid consecutive pleuritic symptoms 37

University of Pennsylvania, . . 38

A new operation for the cure of varicocele. By J. Pancoast, M. D. . 38

*Wills' Hospital.* Clinic of Dr. Isaac Parrish,

Case 1. Scrofulous conjunctivitis — ulcers of the cornea—photophobia, &c. 39

*Bibliographical Notices.*

Report of the Pennsylvania Hospital for the Insane, for the year 1842. By Thomas S. Kirkbride, M. D., Physician to the Institution. Philadelphia: 1843. 8vo. pp. 59, . . . . 41

On a Variety of False Aneurism. By Robert Liston, F. R. S., Surgeon to University College Hospital, Professor of Clinical Surgery, &c. &c. London: 8vo. pp. 39, . . . . 42

Treatise on the Dental Art, founded on Actual Experience. Illustrated by 241 figures in Lithography, and 44 Wood Cuts. By F. Maury, Dentist of the Royal Polytechnic School. Translated from the French, with Notes and Additions. By J. B. Savier, Doctor in Dental Surgery. Philadelphia: 1843. 8vo. pp. 285. . 43

Jefferson Medical College, . . 43

*Retrospect of the Medical Sciences.*

Successful operation for strangulated hernia at the age of 107, . . 44

Suette Miliare, . . . . . . . 44

Physiology of Menstruation, . . 44

The theory of Cells opposed, . . 45

Lactucarium, . . . . . . . 45

Sudden deaths at Strasbourg, . . 46

The Blindness caused by Vitriol, . 46

Carbonic Acid exhaled during Respiration, . . . . . . . 46

Iodide of Potassium in Atonic Ulcers, 46

Lactate of Quinine, . . . . . 47

Varicocele, . . . . . . 47

Means of rendering the Preservation and Use of the Nitrate of Silver more certain and more easy. By Professor Dumeril, . . . . . . 48

Difference of the Pus-like Globules of the Blood in health from those in disease, . . . . . . . 48

Mode of formation of red Globules, . 48

